# A matter of atrophy: differential impact of brain and spine damage on disability worsening in multiple sclerosis

**DOI:** 10.1007/s00415-021-10576-9

**Published:** 2021-05-03

**Authors:** Serena Ruggieri, Maria Petracca, Laura De Giglio, Francesca De Luca, Costanza Giannì, Flavia Gurreri, Nikolaos Petsas, Silvia Tommasin, Carlo Pozzilli, Patrizia Pantano

**Affiliations:** 1 Department of Human Neurosciences, Sapienza University, Rome, Italy; 2 Neuroimmunology Unit, IRCSS Fondazione Santa Lucia, Rome, Italy; 3 Department of Neurosciences and Reproductive and Odontostomatological Sciences, University of Naples Federico II, Naples, Italy; 4 Neurology Unit, Medicine Department, San Filippo Neri Hospital, Rome, Italy; 5 PhD Program in Behavioral Neuroscience, Sapienza University, Rome, Italy; 6 MS Center, Sant’Andrea Hospital, Rome, Italy; 7 Department of Radiology, IRCCS NEUROMED, Pozzilli, Italy

**Keywords:** Multiple sclerosis, Brain atrophy, Spinal cord atrophy, Disability worsening

## Abstract

As atrophy represents the most relevant driver of progression in multiple sclerosis (MS), we investigated the impact of different patterns of brain and spinal cord atrophy on disability worsening in MS. We acquired clinical and MRI data from 90 patients with relapsing–remitting MS and 24 healthy controls (HC). Clinical progression at follow-up (mean 3.7 years) was defined according to the Expanded Disability Status Scale-Plus. Brain and spinal cord volumes were computed on MRI brain scans. After normalizing each participants’ brain and spine volume to the mean of the HC, z-score cut-offs were applied to separate pathologically atrophic from normal brain and spine volumes (accepting a 2.5% error probability). Accordingly, MS patients were classified into four groups (Group I: no brain or spinal cord atrophy *N* = 40, Group II: brain atrophy/no spinal cord atrophy *N* = 11, Group III: no brain atrophy/ spinal cord atrophy *N* = 32, Group IV: both brain and spinal cord atrophy *N* = 7). All patients’ groups showed significantly lower brain volume than HC (*p* < 0.0001). Group III and IV showed lower spine volume than HC (*p* < 0.0001 for both). Higher brain lesion load was identified in Group II (*p* = 0.049) and Group IV (*p* = 0.023) vs Group I, and in Group IV (*p* = 0.048) vs Group III. Spinal cord atrophy (OR = 3.75, *p* = 0.018) and brain + spinal cord atrophy (OR = 5.71, *p* = 0.046) were significant predictors of disability progression. The presence of concomitant brain and spinal cord atrophy is the strongest correlate of progression over time. Isolated spinal cord atrophy exerts a similar effect, confirming the leading role of spinal cord atrophy in the determination of motor disability.

## Introduction

Multiple Sclerosis (MS) is one of the most common causes of disability in young adults and leads to progressive accumulation of motor and cognitive deficits, with a severe impact on quality of life [1]. In particular, motor deficit plays an important role in global clinical disability since it causes significant disorganization of an individual’s function, with deleterious effects on daily activities [2]. Over the years a great effort has been put into the characterization of the neural correlates of motor impairment in MS, as well as into the identification of predictive markers of clinical disability. As a result, it has been established that the clinical expression of brain damage in MS varies according to multiple factors and that it is poorly explained by conventional Magnetic Resonance Imaging (MRI) findings [3, 4]. Although a full characterization of the imaging correlates of motor impairment in MS is still lacking, several works have highlighted how the rate of cerebral atrophy is able to add information on the progression of disability in patients with MS [5], and how atrophy and lesion volumes present a complementary prognostic value in predicting long term disability [6]. Additionally, infratentorial and spinal cord damage, as well as damage to the corticospinal tract, explain to a varying degree both walking and hand dexterity impairment in MS [7]. This evidence is in line with several works across literature, showing an association between decreased spinal cord volume and disability in MS [8, 9]. In particular, spinal cord atrophy occurs frequently in MS patients and it is present from the early stages of the disease [10], although it is more pronounced in progressive patients compared to individuals with either radiologically isolated syndrome or relapsing–remitting (RR) MS [11].

Given the fundamental role of the spinal cord in upper and lower limb motor function, it is intuitive to understand how pathological processes involving this tiny but complex structure determine a high level of disability in MS patients, as captured by different clinical measures depicting motor impairment. Accordingly, reduction of spinal cord volume is a strong predictor of physical disability and disease progression over time, indicating that spinal cord monitoring can contribute to the estimation of disease activity and severity [12]. Indeed, the association between reduced spinal cord area and increased motor impairment seems to occur independently of brain atrophy or at least to a different extent or rate [13]. In addition, an association between spinal cord atrophy and reduced peripapillary retinal nerve fiber layer thickness has been detected [14], suggesting that spinal cord volume loss might partially reflect global pathological alterations and not only focal damage of long tracts [15].

Given the recognized role of brain and spinal cord atrophy in driving disability, we hypothesized that patients presenting atrophy in both districts (i.e. brain and spinal cord) would be more prone to develop future disability compared to those who present preferential involvement of one of the two compartments. To explore this hypothesis, we evaluated the impact of different patterns of brain and spinal cord atrophy on disability worsening in a cohort of patients with RR MS.

## Materials and methods

### Study participants

Patients with clinically defined MS according to McDonald Criteria [16] and RR phenotype [17] underwent MRI as part of research studies ongoing at our center from 2010 to 2013. For the cross-sectional analysis, we retrospectively collected Expanded Disability Status Scale (EDSS), Timed 25-foot walk test (25-FWT) and Nine Hole Peg Test (9HPT) from clinical evaluations performed within one week of MRI. For longitudinal purposes, from March 2015 to July 2015, a new clinical evaluation was performed. Mean follow-up (FU) interval was 3.70 ± 1.44 years. At both time-points the following information was collected: age, time since first symptoms, disease duration, clinical phenotype and specific treatment for MS (yes/no).

We reviewed all included patients’ clinical history and confirmed the MS diagnosis according to more recent criteria [18].

A cohort of age- and sex-matched healthy control (HC), who underwent MRI in our center during the same period of time, was selected.

The study was conducted after institutional ethical committee approval and was in accordance with the declaration of Helsinki. Written informed consent was obtained from all subjects.

None of the MS participants had experienced clinical relapses within three months from participation at both baseline and FU.

### MRI acquisition protocol

The imaging study was performed with a 3.0-T MR unit (Verio; Siemens, Erlangen, Germany). The manufacturer’s 12-channel head coil designed for parallel imaging (generalized autocalibrating partially parallel acquisition- GRAPPA) was used for radiofrequency signal reception. A multiplanar T1-weighted localizer image with section orientation parallel to the subcallosal line was acquired at the beginning of each MR imaging examination. Brain MRI imaging protocol included the following sequences for all subjects: 1. High-resolution 3 dimensional T1-weighted (3D-T1) Magnetization Prepared Rapid Acquisition Gradient Echo sequence: TR = 1900 ms; TE = 2.93 ms; flip angle = 9°; field of view [FOV] = 260 mm; matrix = 256 × 256; 176 sagittal slices 1 mm thick; no gap; 2. Dual turbo spin-echo, proton density (PD) and T2-weighted images: TR = 3320 ms; TE1 = 10 ms; TE2 = 103 ms; FOV = 220 mm; matrix = 384 × 384; 25 axial slices 4 mm thick; 30% gap. For the cervical spinal cord we used: 3. T2-weighted sequence: TR = 3800 ms; TE = 123.0 m; FOV = 280 mm; matrix = 288 × 448; flip angle = 160°, 13 sagittal slices 3 mm thick, 10% gap; 4. STIR T2-weighted sequence: TR = 4000 ms; TE = 55.0 m; FOV = 250 mm; matrix = 240 × 320; flip angle = 150°, 13 sagittal slices 3 mm thick, 10% gap.

### MRI imaging analysis

Image data were processed on Linux workstations using the FMRIB Software Library 5.9 package (FMRIB Image Analysis Group, Oxford, England, http://www.fmrib.ox.ac.uk/ fsl) and Jim 6.0 software (Xinapse Systems, Essex, England; http:// www.xinapse.com). The analysis has been carried out in 2019.

### Brain and lesion volumes

Lesion volumes were obtained using a semi-automated technique based on local thresholding with the Jim software. Lesions were segmented on PD images, while T2-weighted images were used to increase the confidence level in lesion identification by two neurologists (SR, LDG). Lesion volumes yielded the following data for every subject: a quantification of the lesion burden (total lesion volume—LV) and a binary lesion mask needed for the volumetric analysis, which was co-registered onto the 3D-T1 images. Brain volumes were normalized to standard space MNI reference image to avoid head-size dependencies, and measured using SIENAx [19] on lesion filled brain images [20] to obtain normalized brain volume (NBV).

### Spinal cord volume

Spinal cord volume was measured on the brain 3D-T1 images from C2 to C3 using a semi-automatic segmentation method (Jim version 6.0; Xinapse Systems, Essex, England). First, the sagittal 3D-T1 was reformatted and resampled axially to a 1-mm slice thickness, with the image plane perpendicular to the cord at the C2/C3 disk level. On this image, a marker was placed at the level of the most inferior slice passing centrally through the C2/C3 disk. Then, moving back up, two markers were placed after every five slices, until the fifteenth slice from the first maker was reached. An active surface method was then applied, using the markers of the cord centerline as input. An automatic calculation of spine volume was eventually obtained. To compensate for the biological variation of structural measurements, unrelated to disease effects, the raw volume was subsequently normalized dividing it by the number of slices [21]. The presence and number of spinal cord lesions from C1 to C3 level was assessed on spinal cord MRI T2-weighted and STIR images.

### Atrophy cut-offs definition and patients’ classification

Individual NBV and spinal cord volume were normalized to the mean and the standard deviation values of the HC group thus obtaining z-scores. To classify each patient according to a specific atrophy pattern, we followed the procedure described in Raji et al. [22]. Briefly, in the HC cohort, given the normal distribution of brain and spinal cord volumes, 95% of the brain volume values were located within the area of the mean ± 1.77 standard deviation, while 95% of the spinal cord volume values were located within the area of the mean ± 1.87 standard deviation. Only 5% of the brain/spinal cord volume values were expected to be larger or smaller. Therefore, we assumed that z-scores below  – 1.77 and  – 1.87 represented, respectively, a significant brain/spinal cord volume reduction with an error probability of 2.5% at most. These z-score cut-offs were consequently applied to group individual MS patients based on their brain and spinal cord volumes into the following classes:

Group I: no brain or spinal cord atrophy (z-scores greater than  – 1.77 and  – 1.87, respectively);

Group II: brain atrophy (z-scores lower than  – 1.77), no spinal cord atrophy (z-scores greater than  – 1.87);

Group III: no brain atrophy (z-scores greater than  –  1.77), spinal cord atrophy (z-scores lower than  – 1.87);

Group IV: both brain and spinal cord atrophy (z-scores lower than  –  1.77 and  – 1.87, respectively).

### Statistical analysis

Statistical analyses were performed in SPSS 25.0, with a significance level α = 0.05.

Differences in age and sex between patients and controls were tested via *t*-test and Fisher test, respectively. Pearson chi-square test was used to test differences in sex between the 4 groups. Analysis of variance (ANOVA) was used to test differences in age, disease duration, clinical parameters/lesion loads at baseline and follow-up interval between the 4 groups with post-hoc analysis accounting for multiple comparisons (Bonferroni). Analysis of covariance (ANCOVA), accounting for age and gender, was used to test differences in brain and spinal cord volumes between HC and the 4 patients’ groups, with post-hoc analysis accounting for multiple comparisons (Bonferroni).

Finally, the relationship between atrophy groups and disease progression was tested via logistic regression, entering atrophy classes as independent variable and progression as dependent variable. Progression was defined for each clinical measure as follow: (i) EDSS: increase of 1.5 points for patients with a baseline EDSS score of 0, increase of 1 point for patients with baseline EDSS score from 1.0 to 5.0, and increase of 0.5 points for patients with baseline EDSS score equal or higher to 5.5 [23]; (ii) 9HTP and (iii) 25-FWT: > 20% increase from baseline to FU [24]. Patients were then divided into two groups (progressed versus clinically stable) according to a progression measure known as “EDSS-Plus,” described as progression on ⩾1 of the 3 components (EDSS, 25-FWT, and/or 9HPT) [25].

## Results

### Cross-sectional analysis

**Fig. 1:**
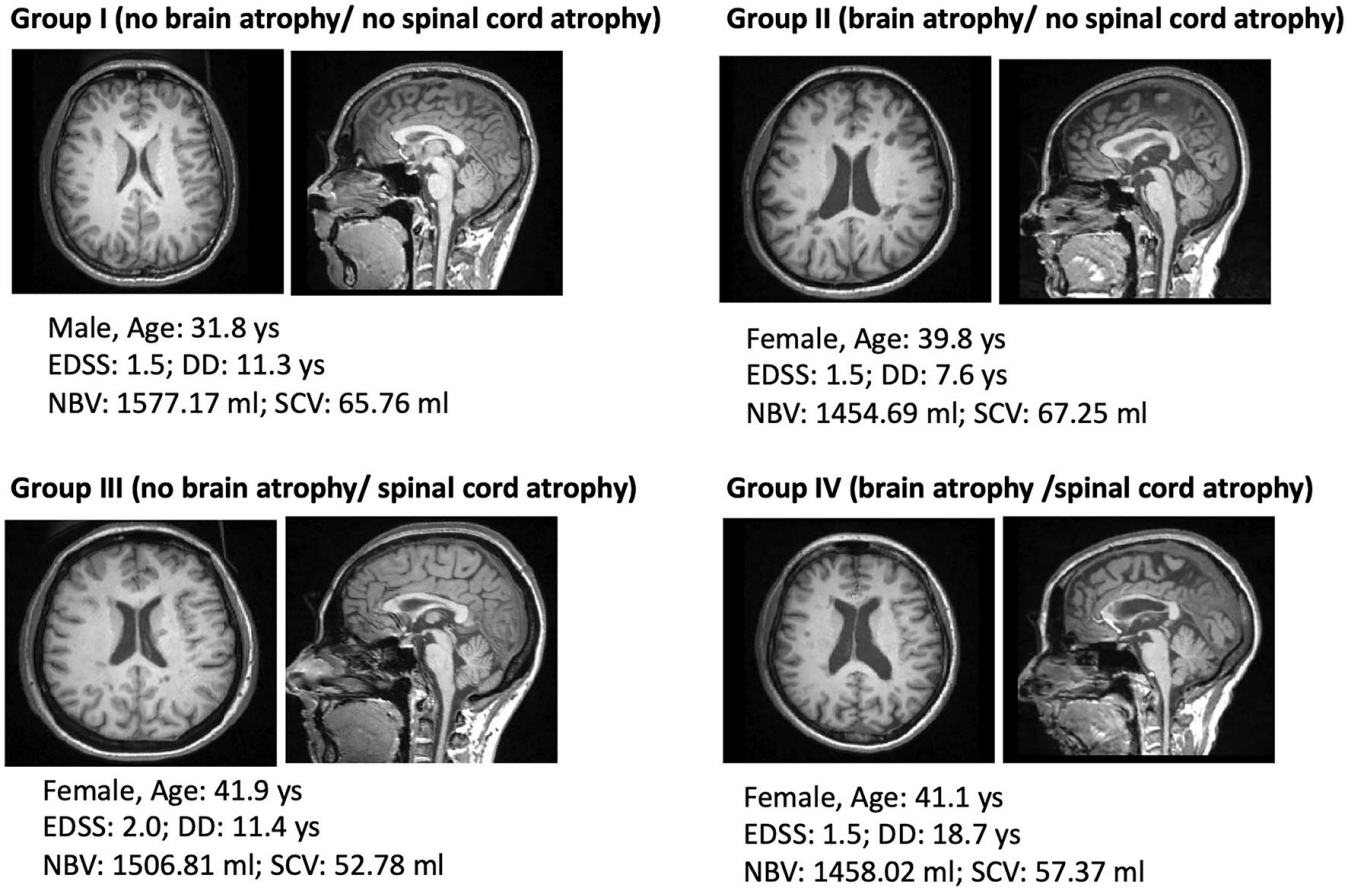
Representative high-resolution 3DT1-MPRAGE showing the distinct atrophy patterns. 3DT1-MPRAGE axial and sagittal slices from four patients, representative of the distinct atrophy patterns, are shown. Group I (no brain atrophy/ no spinal cord atrophy); Group II (brain atrophy/ no spinal cord atrophy); Group III (no brain atrophy/ spinal cord atrophy); Group IV (brain atrophy/ spinal cord atrophy). *DD* disease duration, *EDSS* expanded disability status scale, *NBV* normalized brain volume, *SCV* spinal cord volume

**Fig. 2:**
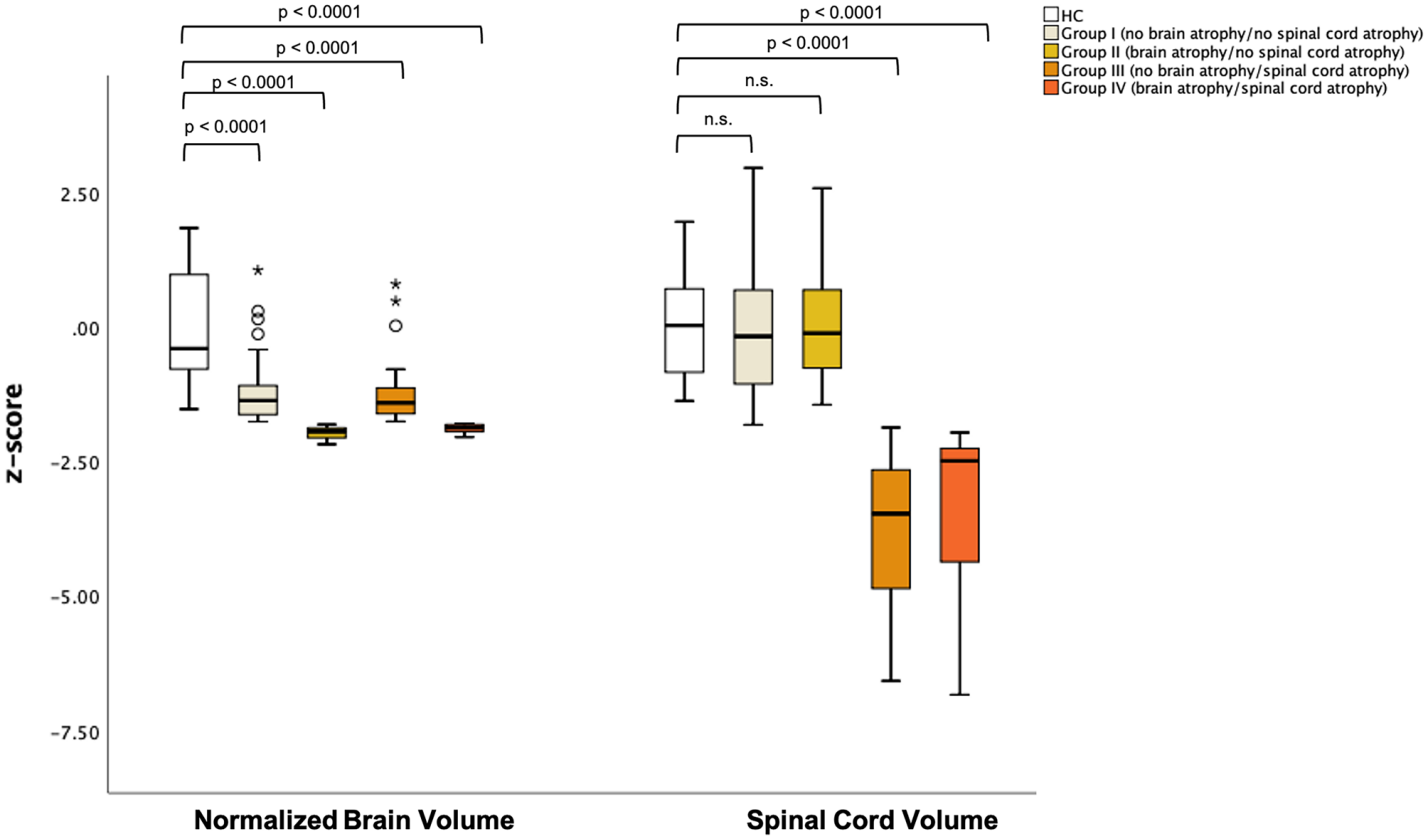
Brain and spinal cord volume z-scores for patients and control. Boxplots showing the 25% to 75% values (boxes) ± 95% values (whiskers), median values (horizontal lines within boxes) of mean Normalized Brain Volume z-score and Spinal Cord Volume z-score value distribution among healthy controls and patients with relapsing–remitting multiple sclerosis. Displayed *p*-values refer to between-group analysis of covariance (ANCOVA), accounting for age and gender, Bonferroni corrected for multiple comparisons. *HC* healthy controls

Ninety MS patients and 24 HC, showing no significant difference in terms of age (35.65 ± 7.78 years vs 32.45 ± 6.41, *p* = 0.07) and sex (67 F vs 18 F, *p* = 0.592), participated in the study. In the patients group 40 subjects were classified in Group I (no brain or spinal cord atrophy), 11 were categorized in Group II (brain atrophy/no spinal cord atrophy), 32 were identified as belonging to Group III (no brain atrophy/spinal cord atrophy) and 7 were included in Group IV (brain and spinal cord atrophy) according to their individual brain and spinal cord volume z-score (Fig. 1). Boxplots displaying mean values for brain and spinal cord volume z-scores in the HC and in the 4 patients’ group are displayed in Fig. 2. All patients’ groups showed significantly lower NBV z-score than HC (*p* < 0.0001 for all), while only Group III and Group IV showed lower spinal cord volume z-score than HC (*p* < 0.0001 for both). Differences in clinical and MRI parameters among the 4 groups are shown in Table 1. No differences emerged between atrophy classes in terms of FU interval or disease duration. From a clinical perspective, patients in Group IV (brain and spinal cord atrophy) presented worse 9HPT performance at baseline than patients in Group I (no brain or spinal cord atrophy) (24.96 ± 14.03 s. vs 18.87 ± 2.44, *p* = 0.015). Patients classified as Group II (brain atrophy/no spinal cord atrophy) and Group IV (brain and spinal cord atrophy) presented higher brain lesion load than patients classified in Group I (no brain or spinal cord atrophy) (respectively, 7.92 ± 8.59 ml and 9.31 ± 6.24 ml vs 3.54 ± 4.37, *p* = 0.049 and *p* = 0.023), and patients classified as Group IV(brain and spinal cord atrophy) presented higher brain lesion load than patients belonging to Group III (no brain atrophy/spinal cord atrophy) (9.31 ± 6.24 ml vs 3.93 ± 2.68, *p* = 0.048). No significant between group difference was identified for spinal cord lesion load.

**Table 1:** Baseline demographics, clinical features and MRI features of the study population

	Whole Cohort (*n* = 90)	Group I (*n* = 40)	Group II (*n* = 11)	Group III (*n* = 32)	Group IV (*n* = 7)	*p*^a^	p^b^	p^c^	p^d^	p^e^	p^f^
Age (yrs)	35.64 ± 7.77	33.97 ± 7.18	34.89 ± 8.59	36.95 ± 8.31	40.38 ± 5.13	1	0.631	0.267	1	0.853	1
DD (yrs)	6.81 ± 6.85	5.29 ± 7.19	8.60 ± 5.14	7.24 ± 7.02	9.86 ± 5.25	0.941	1	0.627	1	1	1
EDSS, median (range)	1.5 (0–4.5)	1.5 (0–4.0)	1.5 (1.0–2.5)	2.0 (0–4.5)	2.5 (1.5–4.5)	1	0.145	0.478	0.365	0.533	1
9HPT (sec)	20.12 ± 4.98	18.86 ± 2.43	19.24 ± 3.37	20.95 ± 3.78	24.96 ± 14.30	1	0.409	**0.015**	1	0.91	0.283
25-FWT (sec)	6.63 ± 3.58	5.98 ± 1.76	6.37 ± 2.64	7.51 ± 5.38	6.80 ± 2.19	1	0.471	1	1	1	1
FU (yrs)	3.71 ± 1.43	3.85 ± 1.44	2.83 ± 1.40	3.93 ± 1.41	3.38 ± 1.13	0.217	1	1	0.168	1	1
Brain LV (ml)	4.66 ± 5.05	3.53 ± 4.36	7.91 ± 8.59	3.93 ± 2.67	9.31 ± 6.24	**0.049**	1	**0.023**	0.111	1	**0.048**
Spine LC	0.94 ± 0.80	0.85 ± 0.87	0.45 ± 0.52	1.10 ± 0.72	1.50 ± 0.83	0.894	1	0.344	0.125	0.058	1
NBV (ml)	1516.42 ± 69.24	1534.24 ± 68.79	1447.46 ± 13.90	1531.02 ± 67.75	1456.21 ± 10.55	**0.010**	1	0.341	**0.010**	1	0.290
SCV (ml)	61.03 ± 9.08	67.36 ± 5.06	67.81 ± 4.88	52.50 ± 5.34	53.18 ± 4.12	1	** < 0.001**	** < 0.001**	** < 0.001**	** < 0.001**	1

Values are expressed as mean ± standard deviation, unless otherwise indicated

Group I: no brain atrophy/ no spinal cord atrophy; Group II: brain atrophy/ no spinal cord atrophy; Group III: no brain atrophy/ spinal cord atrophy; Group IV: brain atrophy/ spinal cord atrophy

Analysis of variance (ANOVA), p refers to post-hoc analysis accounting for multiple comparisons (Bonferroni). Normalized Brain Volume and Spinal Cord Volume comparison were adjusted for age and gender. *p*^a^ Group I vs Group II; *p*^b^ Group I vs ‘Group III; *p*^c^ Group I vs Group IV; *p*^d^ Group II vs Group III; *p*^e^ Group II vs Group IV; *p*^f^ Group III vs Group IV. Significant differences are highlighted in bold

Abbreviations: 9HPT = 9-hole peg test, 25- FWT: 25-foot walking test, DD: disease duration, EDSS: Expanded Disability Status Scale, FU: follow-up, LV: lesion volume, LC: lesion count, NBV: normalized brain volume, SCV: spinal cord volume

### Longitudinal analysis

Seven patients were lost at FU. Of the remaining 83 patients, 29 showed disability progression. In particular, 5 patients presented with worsening of 9HPT, 2 showed an increase of 25-FWT score and 11 a progression in EDSS score, while the remaining 11 patients showed a deterioration in more than one clinical outcome.

The regression model including atrophy classes significantly predicted clinical progression (Nagelkerke R Square = 0.129, *p* = 0.043), with a significant predictive role identified for spinal cord atrophy (OR = 3.75, 95% IC: 1.26—11.17, *p* = 0.018) and brain + spinal cord atrophy (OR = 5.71, 95% IC: 1.03  – 31.53, *p* = 0.046) in comparison with the class showing no atrophy.

## Discussion

In the present study, we investigated whether defining a specific atrophy pattern on a single MRI acquisition could assist in predicting the development of motor disability in a group of RR MS patients within the first 10 years of the disease. The role of brain and spinal cord atrophy in driving disease progression has been widely investigated in MS, and the question of their relative contribution to disability is not new [26]. Indeed, both in relapse and progressive onset MS, cross-sectional investigations suggest that brain and spinal cord atrophy independently contribute to medium (10 years) [14, 27, 28] and long-term (> 15 years) physical disability [9, 29–32]. Longitudinal findings support the relevance of the ﻿annual rate of spinal cord volume loss, rather than brain metrics, in predicting ﻿the annual EDSS score change over 6 years [12]. Additionally, baseline measures of cervical atrophy appear to be more significant than brain atrophy in predicting disability progression at 2 years [33, 34]. Here, building on these previous findings, we identified 4 atrophy patterns based on cross-sectional MRI data, and confirmed that patients showing spinal cord atrophy indeed presented a high risk of short-term (3.7 years) disability progression. Additionally, patients showing atrophy of both brain and spinal cord presented an even higher progression risk, suggesting that atrophy occurring in the two compartments exerts a cumulative effect on disability progression, which is in line with the concept that atrophy accrual in the brain and spinal cord is underpinned by different and at least partially independent neurodegenerative mechanisms [13, 35]. These results further underline how atrophy constitutes a valuable proxy of irreversible damage and predictor of consequent progression even over the short-term and in the RR phase, when the inflammatory component of the disease still overshadows neurodegeneration.

Lately, atrophy exploration in MS has shifted towards the identification of specific patterns of atrophy development, which could provide a more effective tool to capture the nuanced aspects of disability progression, partially explaining the large individual variability observed. Studies investigating atrophy patterns have mainly focused on longitudinal analysis of brain volumes [36–41] with few investigations exploring spinal cord volumes over time [42] or both brain and spinal cord atrophy [43]. Among these, a small study comparing brain and spinal cord atrophy patterns in ﻿neuromyelitis optica (NMO) and MS concluded that while spinal cord atrophy rate was a significant driver of disability progression in NMO, brain atrophy played a preponderant role in MS [43]. These results, which are in apparent disagreement with the body of literature on spine damage in MS, might be explained by the small sample size and the short FU period (1 year), which might have been sufficient to capture spinal cord modifications in NMO but not in MS. Indeed, in a recent work [44] both association and multivariable analyses showed that cervical spinal cord gray matter lesion and volumetric measures explained more variance in disability parameters than global or tissue-specific computation in the brain, yielding to the conclusion that determination of cervical spinal cord lesions and atrophy might be more crucial than brain measures in explaining physical disability in MS [45].

While most of the abovementioned studies on atrophy patterns adopted a data driven identification of different classes based on the rate of change over time, our definition of atrophy patterns is based on a hypothesis-driven classification of cross-sectional data, in the attempt to clarify if a single time-point assessment could provide meaningful information on disease progression. Even though our sample size was relatively small, we were able to identify four groups of patients well balanced in terms of age, gender and disease duration. Interestingly, the four groups we identified were substantially homogeneous for spine but not for brain lesion loads, which is in line with the concept that, while brain atrophy is partially dependent from global lesion load, spine atrophy appears to be a relatively ﻿independent process [30, 46, 47]. Additionally, the four groups did not show substantial differences in terms of clinical scores at baseline, with the exception, in patients with both brain and spinal cord atrophy, of worse manual dexterity in comparison with patients with no atrophy. These findings might point to a higher sensitivity of 9HPT towards damage of multiple districts in comparison to the other clinical measures explored, that, being heavily weighted towards or measuring directly ambulation, would be more affected by spinal cord damage and, in particular, spinal cord lesions, which might explain the lack of differences in EDSS and 25-FWT observed among the 4 groups at baseline. Even though atrophy represents a continuum, as underlined by the finding that all patients’ group, including the one labelled as presenting no atrophy (Group I), showed a mean NBV lower than HC, our choice to categorize patients in classes based on the binary assessment atrophy/no atrophy in brain and spinal cord compartments represents a useful simplification that has provided relevant information. In fact, although we are still far from the translation of atrophy measurement in general and spine atrophy in particular into clinical practice, the relevance of brain and spinal cord atrophy as predictors of MS evolution is undisputed [15] and our findings add to the body of literature suggesting that spinal cord atrophy represents a valuable tool to define and predict MS severity independently from brain atrophy, hinting to the possibility of defining a clinically meaningful atrophy pattern relying on single time-point data.

Additionally, while previous studies supporting the role of baseline measures of brain and spinal cord atrophy as predictors of future disability have been conducted on mixed samples including both relapsing and progressive patients [33, 34], we focused on RR patients, proving the validity of such metrics in this population over a short period of time.

Our work is not without limitations. One that can be pointed out is the usage of brain scans instead of a dedicated spine acquisition to measure spinal cord volume. However, recent works have suggested the possibility to calculate spinal cord atrophy using brain volumetric images [48, 49] in order to save time, to minimize cost and to lessen the amount of motion artifacts. The brain 3DT1 images used in this work fully covered our region of interest, i.e. the upper cervical region. Indeed, the most common levels where spinal cord volume is measured are C1–C2 and C2–C3, since this region is less affected by movement artefacts, leads to the most sensible results, and guarantees optimal clinical correlates [50]. Further supporting our choice, a recent work from MAGNIMS group has confirmed the similarity in terms of reproducibility and sensitivity between spinal cord area measured using specific volumetric sequences and spinal cord area detected using volumetric brain MRI [51]. A second controversial point could be represented by the definition of disability progression that we adopted. Although the choice to utilize the EDSS-Plus has increased the heterogeneity in our group of progressed patients, we believe that this classification accurately reflects the variability that characterizes MS clinical evolution, and therefore represents a necessary prerequisite to the generalizability of our findings. Eventually, as our hypothesis was centered on the influence of brain and spinal cord atrophy on disability, we focused on the development of motor impairment, thus ignoring the evolution of cognitive deterioration, which is a relevant player in the patients’ global disability status. Of note, even though we acquired our data between 2010 and 2015 and finalized the analysis in 2019, the methodology adopted is still representative of the current research practice in the MS field [44, 52], due to the robustness and reproducibility of the chosen techniques.

Notwithstanding these limitations, our findings suggest that the presence of spinal cord atrophy, alone or in combination with brain atrophy estimated at a single time-point, is a meaningful predictor of short-term progression, and could represent a valuable biomarker once methodological ﻿barriers to the implementation of volumetric measures in clinical practice will be addressed.

## Data Availability

The raw data supporting the conclusions of this manuscript will be made available by the authors, without undue reservation, to any qualified researcher.
